# The inhibitory effect of 7,7″-dimethoxyagastisflavone on the metastasis of melanoma cells via the suppression of F-actin polymerization

**DOI:** 10.18632/oncotarget.10960

**Published:** 2016-07-30

**Authors:** Ching-Min Lin, Yu-Ling Lin, Shu-Yi Ho, Pin-Rong Chen, Yi-Hsuan Tsai, Chen-Han Chung, Chia-Hsiang Hwang, Nu-Man Tsai, Shey-Cherng Tzou, Chun-Yen Ke, Jung Chang, Yi-Lin Chan, Yu-Shan Wang, Kwan-Hwa Chi, Kuang-Wen Liao

**Affiliations:** ^1^ Institute of Molecular Medicine and Bioengineering, National Chiao Tung University, Hsinchu, Taiwan; ^2^ Center for Bioinformatics Research, National Chiao Tung University, Hsinchu, Taiwan; ^3^ Department of Biological Science and Technology, National Chiao Tung University, Hsinchu, Taiwan; ^4^ Yung-Shin Pharmaceutical Industry Co., Ltd., Taichung, Taiwan; ^5^ Department of Medical and Laboratory Biotechnology, Chung Shan Medical University, Taichung, Taiwan; ^6^ Clinical Laboratory, Chung Shan Medical University Hospital, Taichung, Taiwan; ^7^ Department of Life Science, Chinese Culture University, Taichung, Taiwan; ^8^ Department of Radiation Therapy and Oncology, Shin Kong Wu Ho-Su Memorial Hospital, Taipei, Taiwan; ^9^ Graduate Institut of Medicine, College of Medicine, Kaohsiung Medical University, Kaohsiung, Taiwan

**Keywords:** biflavonoid, angiogenesis, metastasis, actin polymerization, cAMP response element-binding protein (CREB)

## Abstract

7,7″-Dimethoxyagastisflavone (DMGF), a biflavonoid isolated from *Taxus* × *media* cv. Hicksii, induces apoptotic and autophagic cell death. However, whether DMGF suppresses tumor metastasis is unclear. The aim of this study was to investigate the anti-metastatic activities of DMGF on the metastatic processes of melanoma cells *in vivo* and *in vitro*. A transwell assay showed that DMGF could effectively attenuate the motility of B16F10 cells, and the results of real-time PCR revealed that DMGF also suppressed the expressions of matrix metalloproteinase-2 (MMP-2). Moreover, DMGF did not influence tube formation but inhibited the migration of endothelial cells. Furthermore, animal models were used to monitor the effects of DMGF on tumor metastasis, and all models showed that DMGF significantly suppressed the metastatic behaviors of B16F10 cells, including intravasation, colonization, and invasion of the lymphatic duct. In addition, DMGF could also reduce the densities of the blood vessels in the tumor area *in vivo*. Further investigation of the molecular mechanisms of anti-metastatic activity revealed that DMGF can down-regulate the levels of key modulators of the Cdc42/Rac1 pathway to interfere in F-actin polymerization and suppress the formation of lamellipodia by reducing the phosphorylation of CREB. These data suggested that DMGF presents anti-metastatic activities in B16F10 melanoma cells. Here, we demonstrated that DMGF can inhibit the metastasis of highly invasive melanoma cancer cells through the down-regulation of F-actin polymerization. Considering these findings, DMGF may be further developed to serve as a chemoprevention drug for patients with metastatic melanoma.

## INTRODUCTION

**Figure 1:**
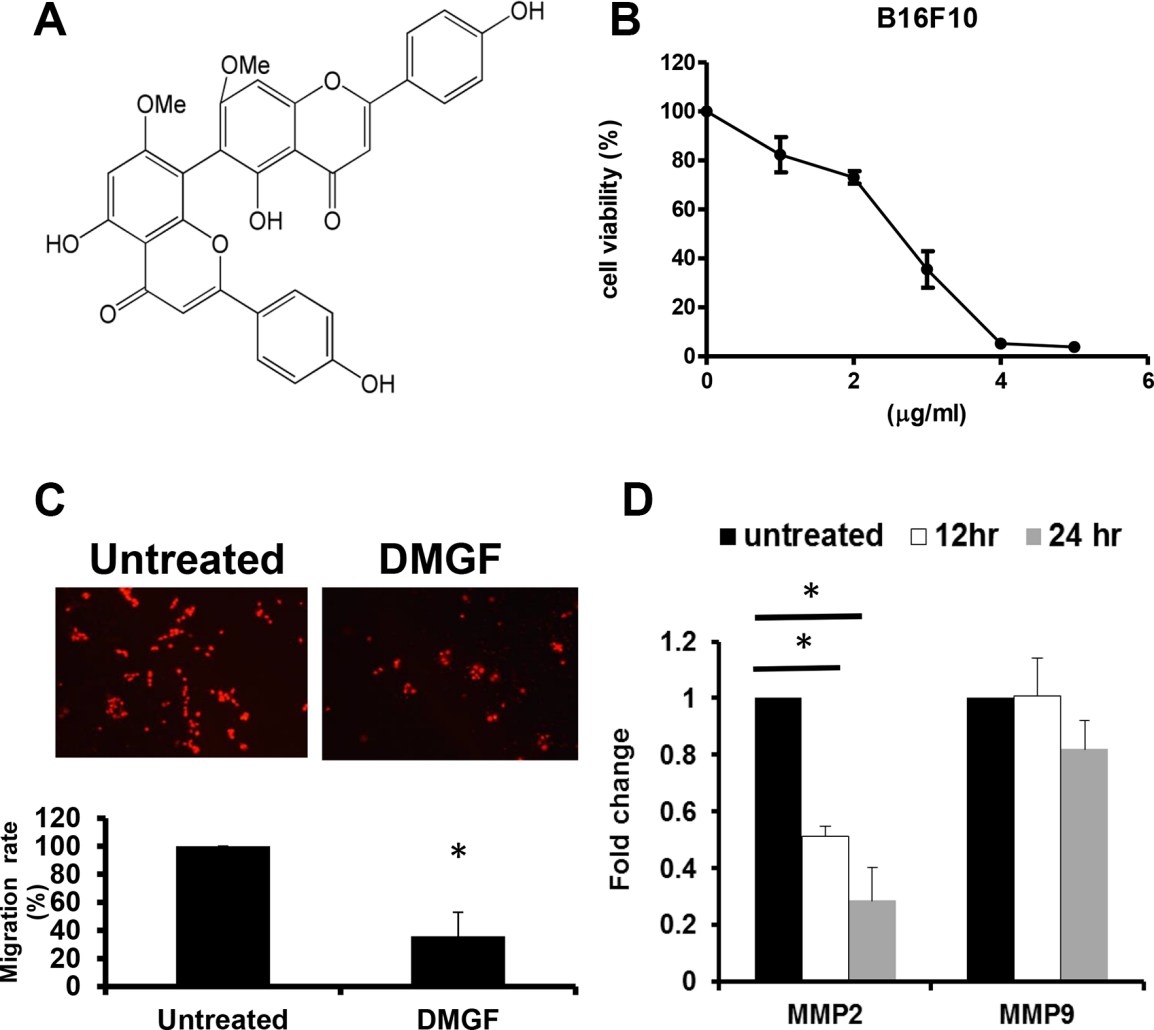
Effects of DMGF for B16F10 melanoma cells (**A**) The structure of DMGF. (**B**) Proliferating B16F10 melanoma cells were treated with various concentrations of DMGF (5, 4, 3, 2 or 1 μg/ml) for 48 h; cell growth was measured by MTT assay. The readings from controls were normalized to 100%, and readings from DMGF-treated cells were expressed as a percentage of controls. (**C**) DMGF (1 μg/ml) inhibited the migration of B16F10 melanoma cells. Migration was quantitated by counting the cells that migrated to the lower side of the filter after fixation and staining (*n* = 3). Migrated cells were counted by an inverted light microscope at 100× magnification. The relative percent of migrated cells was counted and summarized in the bottom panel, and ten random fields were chosen for each count. (**D**) The expression level of MMP2 and MMP9 relative to β-actin was compared between the untreated group and DMGF group in B16F10 melanoma cells using quantitative PCR. (*p < 0.05).

A variety of polyphenolic compounds in dietary and medical plants have been reported to exhibit a variety of biological effects. Among polyphenolic substances, biflavonoids have potent anti-inflammatory, anti-cancer, anti-virus, anti-microbial, vasorelaxant and anticlotting properties [1]. Currently, many biflavonoids exhibit bioactive functions, and the mechanisms of such effects have been explored. For example, the amentoflavone derived from *Selaginella tamariscina* or *Torreya nucifera* has been shown to not only have potent anti-candidal or anti-SARS-coV activities [2, 3], but also exhibits anti-neovascularization by interfering in the interaction of VEGF and VEGFR-1 [4, 5]. In addition, 7,7″-dimethoxyagastisflavone (DMGF, the structure was shown as Figure 1A) has been found to have anti-HSV-1 and -HSV-2 activity [6], and it can cause the cell death via apoptosis or autophagy [7]. Moreover, ginkgetin is a strong NF-κB inhibitor (IC50 = 7.5 μM) [8], and it selectively inhibits the growth of human ovarian adenocarcinoma [9]. Moreover, morelloflavone has anti-oxidative, anti-viral, and anti-inflammatory properties [10], and it was recently shown to inhibit tumor angiogenesis by targeting Rho GTPases and the ERK signaling pathway [11]. Therefore, the biflavonoids in these traditional medical plants are a potential source of new therapies for many human diseases.

Tumor cells metastasize from the original site to other organs, developing and growing to form a new nodule. This process results in dysfunction of the organ and eventual death. Thus, the development of anti-metastasis drugs is important and necessary. However, tumor metastasis is a complex process and consists of a series of sequential steps [12] in which cell movement, extravasation [13], and expressions of matrix metalloproteinases [14] are important processes that result in systemic spread. In addition, angiogenesis is not only required for nourishment and the removal of metabolic wastes from the tumor region, but it also facilitates tumor metastasis. Thus, chemoprotective drugs that can simultaneously inhibit the processes of metastasis and angiogenesis may provide better chemotherapeutic efficiency by inhibiting tumor spread.

In addition to the previous description of DMGF, only one study has described its activity, reporting that it can inhibit the production of aflatoxin by *Aspergillus flavus* [15]. Whether DMGF has other bioactivities in tumor migration is unclear. In this study, the effects of DMGF on metastasis were determined, and the *in vitro* and *in vivo* results indicated that DMGF could decrease the activities of B16F10 cells in metastasis. *In vivo*, DMGF also showed that it could reduce new vessel formation in the tumor area and reduce the abilities of B16F10 cells to metastasize through the new vessel. The results showed that DMGF down-regulated the expressions of the genes associated with the Cdc42/Rac1 pathway to lower the formation of lamellipodia by reducing the phosphorylation of the CREB transcriptional factor. Taken together, these results indicate that DMGF is a good candidate for an anti-metastasis drug.

## RESULTS

### The suppressive potency of DMGF in metastasis *in vitro*

Highly metastatic B16F10 melanoma cells were used to examine the anti-metastatic potency of DMGF. First, the cytotoxicity of DMGF for B16F10 cells was determined, and it was shown to suppress the cell proliferation of B16F10 cells in a dose-dependent manner (Figure 1B). Sequentially, low doses of DMGF were administered to determine whether DMGF could affect the migration of B16F10 cells. Clearly, the migration rate of B16F10 cells was lowered to 35% compared with the untreated group within 6 h (Figure 1C). Furthermore, the elevated levels of MMPs in tumor tissue were correlated with the invasion of cancer cells. Thus, the effects of DMGF on the expressions of MMP genes in tumors were further measured, and the results showed that treatments with DMGF for 12 or 24 h significantly inhibited the MMP-2 expression of B16F10 cells but not MMP-9 compared to untreated B16F10 cells (Figure 1D). Taking these *in vitro* results together revealed that DMGF might suppress the metastasis of tumor cells by suppressing migration and matrix degradation.

### The suppressive potency of DMGF in angiogenesis *in vitro* and *in vivo*

**Figure 2:**
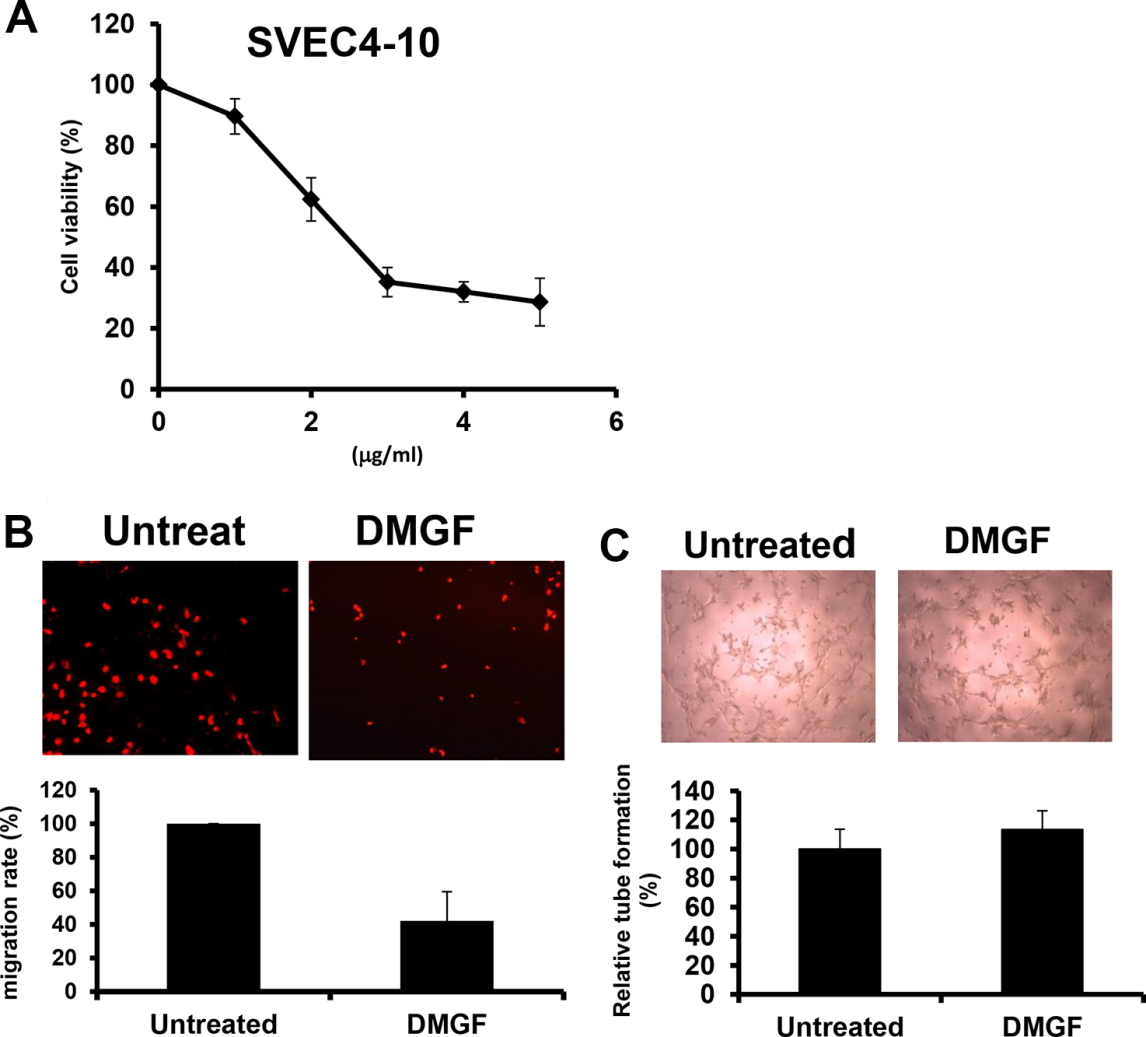
Effects of DMGF on angiogenesis in SVEC4-10 cells (**A**) Proliferating SVEC4-10 cells were treated with various concentrations of DMGF for 48 h; cell growth and viability were measured by MTT assay. (**B**) DMGF inhibited the migration of SVEC4-10 cells. Migration was quantified by counting the cells that migrated to the lower side of the filter after fixation and staining (*n* = 3). Migrated cells were counted by an inverted light microscope at 100× magnification. The relative percent of migrated cells was counted and summarized in the bottom panel, and ten random fields were chosen for each count. (**C**) DMGF (1 μg/ml) had no effect on tube formation of SVEC4-10 cells. Micrographs were taken at 100× magnification. Experiments were repeated three times, and values were the means of triplicates. The relative percent of tubes was counted and summarized in the bottom panel, and ten random fields were chosen for each count. (*p < 0.05).

Tumor metastasis is highly associated with angiogenesis; therefore, the effect of DMGF on angiogenesis was examined *in vitro*. Figure 2A showed that DMGF could inhibit the cell proliferation of endothelial cells. In addition, a transwell assay indicated that low dosages of DMGF also significantly inhibited the migration of endothelial cells (Figure 2B). However, the treatments of DMGF had no effect on the tube formation of mouse SVEC4-10 endothelial cells (Figure 2C). These results indicated that DMGF could only inhibit the early stages of angiogenesis, including cell proliferation and migration for endothelial cells, but not influence the late stage of angiogenesis, including tube formation.

**Figure 3:**
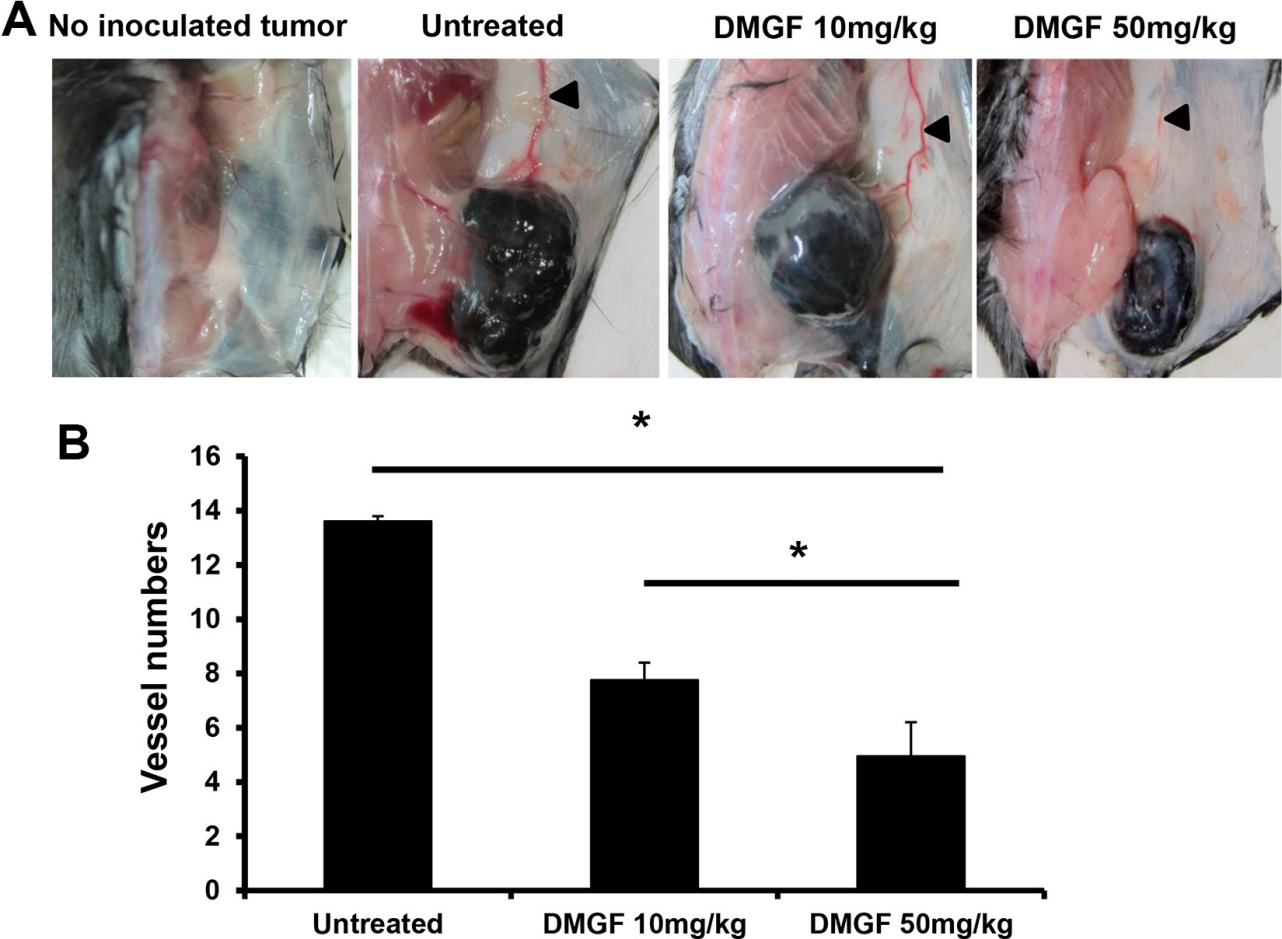
Effect of DMGF on the inhibition of tumor angiogenesis (**A**) Subcutaneous vascularizing of mice. Mice were sacrificed when the tumor volume of the untreated group was up to 2500 mm^3^. Dorsal skin was regularly cut from the mice. (**B**) After H&E staining, the vessels were estimated by counting and averaging 10 fields of every slide under a light microscope at 200× magnification. The numbers of vessels were expressed as the mean ± S.D. Significant differences were reported compared with the PBS treatment group (*p < 0.05).

Furthermore, the *in vivo* effect of DMGF on angiogenesis was determined. When the average volumes of B16/F10 melanoma on the mouse backs were approximately 30 mm^3^, the tumor-bearing mice were treated with or without i.v. injections of DMGF to determine its effects on angiogenesis. Figure 3A revealed that larger new vessels formed near the tumor area in the untreated group, but there was less formation of new vessels near the tumor area in the DMGF-treated group *in vivo*. The sizes of angiogenetic vessels in the dorsal skin were correlated with DMGF dosages (Figure 3A). H&E histological staining further indicated that DMGF treatments lowered the densities of the blood vessels in the tumor area (Figure 3B). Thus, DMGF treatment indeed suppressed angiogenesis *in vivo*. Angiogenesis plays an important role in tumor metastasis. Therefore, the suppression of angiogenesis is one of the mechanisms of metastatic suppression for DMGF.

### The effects of DMGF on tumor metastasis *in vivo*

**Figure 4:**
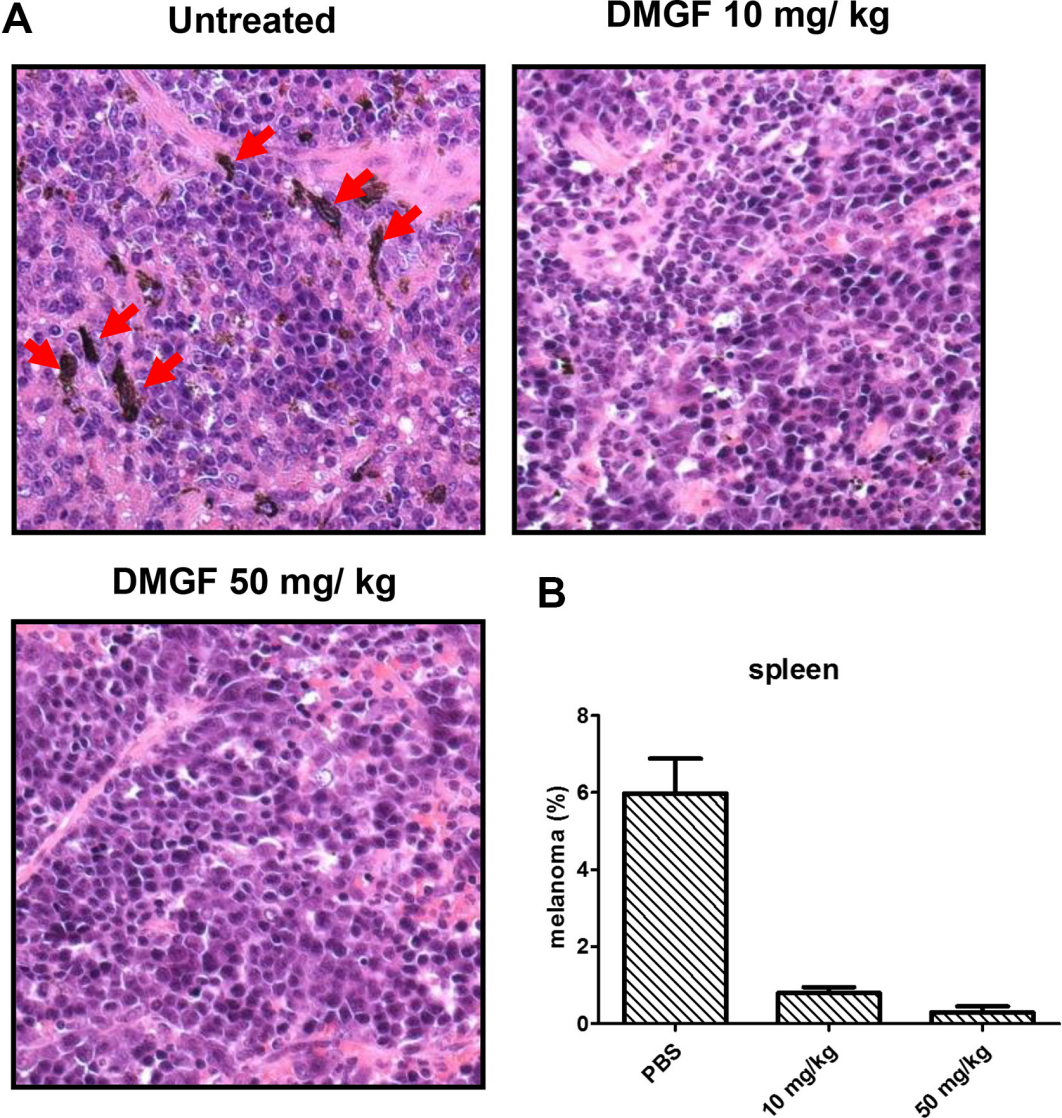
Effect of DMGF on spontaneous metastasis in the spleen (**A**) H&E staining for spontaneous metastasis analysis in the spleen. Mice were sacrificed when the tumor volume of PBS group was up to 2500 mm^3^. The DMGF group contains mice that were *i.v*. injected with 50 mg/kg DMGF. Red arrow indicated the melanoma from hypoderm spontaneously migrated to spleen. (**B**) After H&E staining, the melanoma cells were estimated by counting and averaging 10 fields of every slide under a light microscope at 400× magnification. Significant differences after treatment were found for the DMGF related group compared with the PBS group (*p < 0.05).

With a high metastatic ability, B16F10 cells were used to determine the anti-metastasis ability of DMGF. The tumor-bearing mice were examined to determine whether the tumor cells could spontaneously metastasize from subcutaneous sites to the organs. All organs but the spleen were examined and the melanoma cells were undetectable (results not shown). In the spleen, B16F10 cells spontaneously metastasized from the subcutaneous inoculated sites to the spleen (Figure 4A, right panel) whereas the administration of DMGF decreased the numbers of metastatic melanoma cells in a dose-dependent manner (Figure 4A, middle and left panels). Counting cells under a microscope, DMGF treatments lowered the number of spontaneously metastatic cells in the spleen 5-fold relative to the untreated group (Figure 4B).

**Figure 5:**
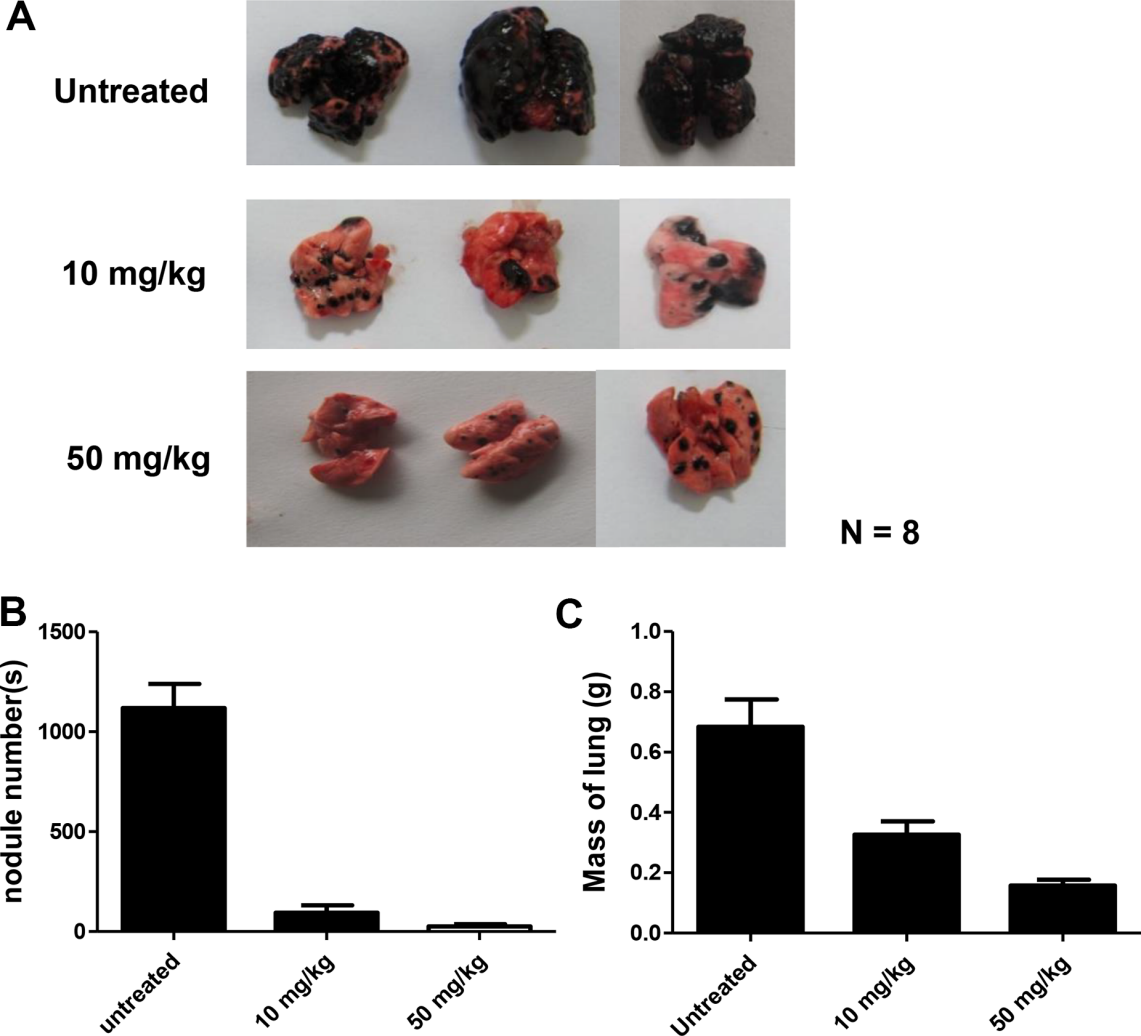
Effect of DMGF on lung metastasis of B16F10 melanoma Representatives of metastatic nodules on the surface of the lungs in C57BL/6JNarl mice induced by injecting 1 × 10^6^ cells intravenously. Mice were sacrificed 19 days after injection. *N* = 8. (**A**) Their lungs were then removed and fixed, metastatic foci at the lung surfaces were photographed. (**B**) The number of foci of lung metastases in each group were detected. (**C**) The mass of the lungs in each group was measured and summarized. Data were collected from two independent experiments. (*p < 0.05).

Metastatic tumor cells have the ability to colonize at new sites through the circulatory system; therefore, B16F10 cells were i.v. injected to examine the effect of DMGF on tumor colonization. At 19 days after tumor injection, B16F10 cells developed serious lung metastasis, and the numbers of metastatic nodules in the lungs of some mice were too large to count (Figure 5A). Consistent with previous results, administration of 10 mg/kg DMGF significantly decreased the colonization of B16F10 cells from the circulation to the lung. Furthermore, 50 mg/kg DMGF displayed stronger effects to inhibit tumor colonization, and even abolish the colonization of metastatic cells in some treated mice (Figure 5A). The nodules of melanoma cells in the lungs in each group were counted, and the lungs were weighed. The results indicated that the DMGF treatments reduced the colonization more than 3-fold (Figure 5B and 5C).

**Figure 6:**
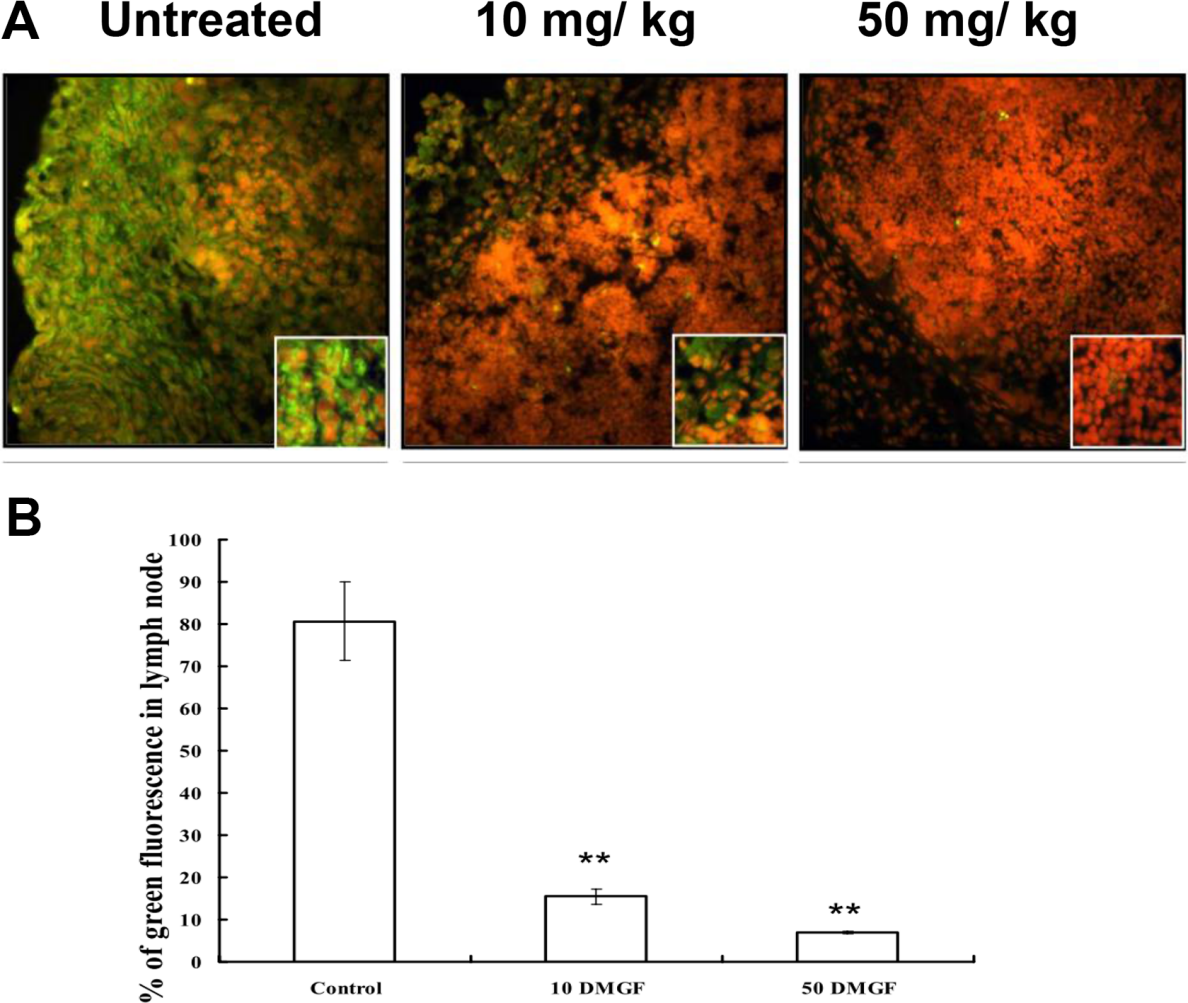
Effect of DMGF on lymph nodule metastasis of melanoma Representation of metastatic cells in the interior area of lymph nodules in C57BL/6JNarl mice induced by injecting 5 × 10^5^ B16F10-EGFP cells approximately 1 cm from the inguinal lymph node. Mice were sacrificed at 9 days after injection. (**A**) Fluorescent metastatic cells in the interior area of inguinal lymph nodes were photographed. (**B**) The ratios of fluorescent metastatic cells in each group were detected and 12 fields of every slide were averaged under a light microscope at 100× magnification. Data were collected from three independent experiments. (*p < 0.05).

Another common pathway for metastatic cells is the lymphoid system. Figure 6A shows that the EGFP-expressing B16F10 cells filled the cortex of the lymph node and invaded the medulla at day 9 after inoculation 1 cm from the inguinal lymph node; DMGF treatments slowed down the migration of tumor cells into the lymph node. After counting, the fluorescent cells occupied 80% of the lymph node. However, DMGF suppressed metastasis to the lymph node in a dose-dependent manner: 10 mg/Kg DMGF lowered lymphoid metastasis from 80% to 16%, and 50 mg/kg DMGF lowered it to below 5% (Figure 6B). These studies clearly revealed that treatment with DMGF efficiently inhibited the B16F10 cells' metastatic ability *in vivo*.

### The mechanisms for the effect of DMGF on metastasis

**Figure 7:**
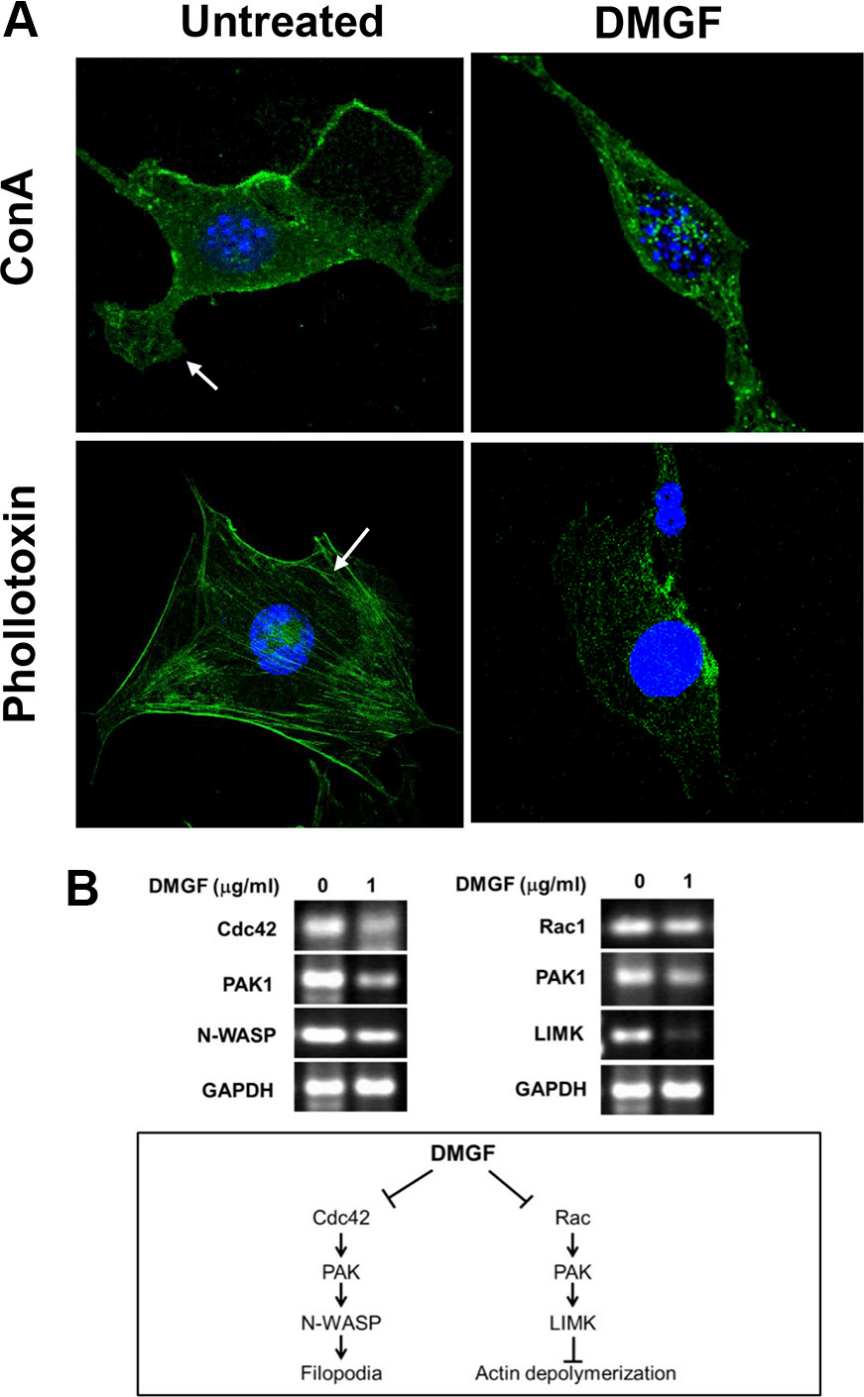
Effect of DMGF on lamellipodia formation and actin assembly (**A**) B16F10 cells were assessed by fluorescence staining with concanavalin A (ConA) conjugated with Alex488 (a substance that specifically detects the cell membrane) or phallotoxin (a substance that specifically detects F-actin). In the untreated group, lamellipodia and stress fibers were present (white arrow, left). Incubation of B16F10 cells with DMGF led to the marked disruption of actin assembly (right). (**B**) Total RNA was extracted in B16F10 cells after DMGF (1 μg/ml) for 12 h and gene expression levels of Cdc42, Rac1, PAK1, N-WASP, and LIMK were assessed by RT-PCR. GAPDH was a loading control.

Metastatic invasion is directly correlated with the formation of lamellipodia, which is caused by the polymerization and depolymerization of actin fibers. Figure 7A revealed that 2.5 mg/ml of DMGF treatments suppressed the formation of lamellipodia (Figure 7A, upper panels). In addition, intracellular actin fiber staining clearly showed that DMGF treatments caused the fragmentation of F-actin (Figure 7A, lower panels). It is well known that the Cdc42 and Rac pathway can regulate actin polymerization or depolymerization to influence the formation of lamellipodia. Figure 7B shows that DMGF treatments suppressed the expression of genes associated with the formation of lamellipodia, including Cdc42, PAK1 and N-WASP. For actin depolymerization, DMGF dramatically suppressed the expression of LIMK, and also decreased the expression of PAK1. Taken together, these results indicated that DMGF could enhance actin depolymerization and suppress the formation of lamellipodia by lowering the expression of the relative genes.

**Figure 8:**
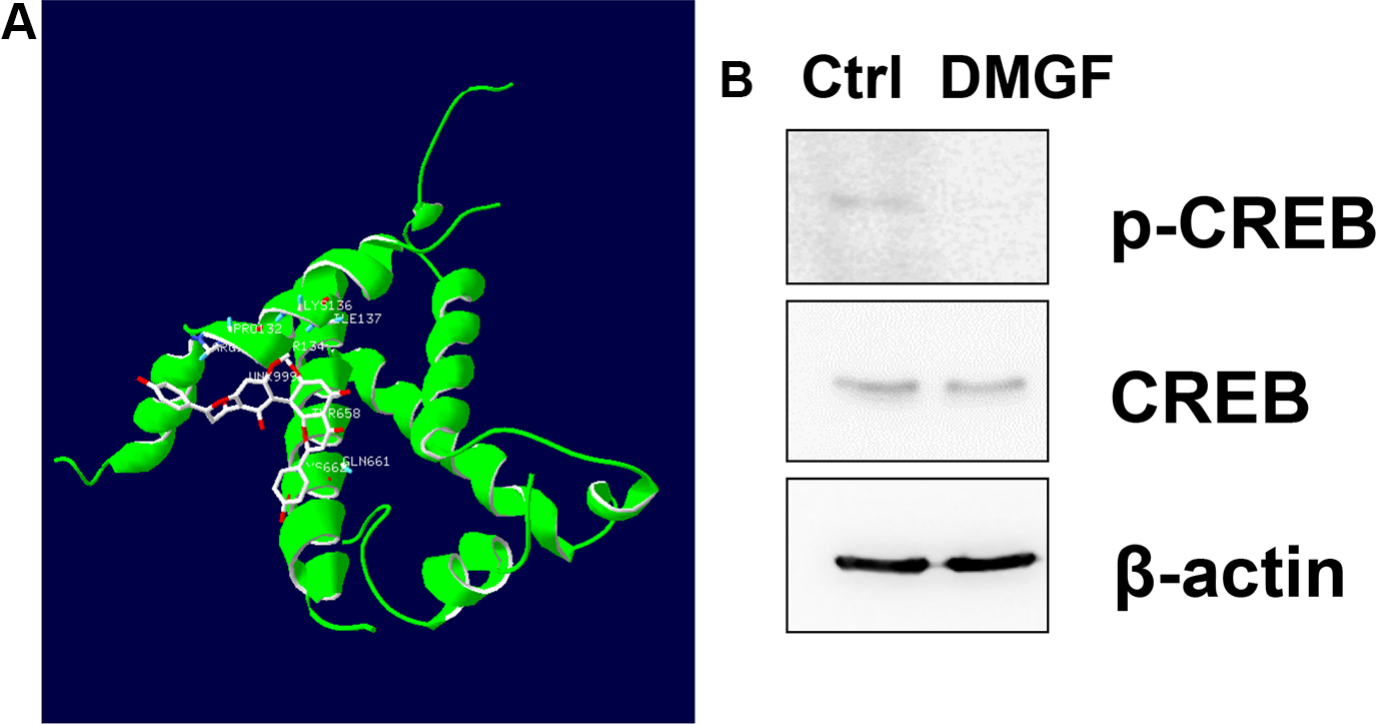
Interactions of CREB and DMGF (**A**) CREB (PDB code: 2LXT) was docked with DMGF molecule by iGEMDOCK software. The structure of CREB protein or DMGF was displayed in green or white color, respectively. (**B**) With or without DMGF treatment, the lysates of B16F10 cells were analyzed by anti-CREB or anti-pCREB antibodies, respectively. Detection of anti-β-actin is as loading control.

Gene expression is driven by the promoter located upstream of the gene. By analyzing the promoters of these DMGF-effective genes for the binding sites of transcriptional factors listed on GeneCards (http://www.genecards.org), the results showed that only one transcriptional factor binding site is displayed on all 5 promoter sequences of these genes–CREB. According to this information, we examined the interaction of DMGF and CREB by bioinformatics tool and the results predicted DMGF would interact with several residuals in CREB (PDB code: 2LXT) including Arg131, Pro132, Tyr134, Lys136, Ile137, Tyr658, Gln661 and Lys662 (Figure 8A). Therefore, the phosphorylation on Ser133 of CREB may be interfered by DMGF. Consecutively, we further examined the CREB protein expression level and its phosphorylation after DMGF treatment. The results showed that DMGF did not notably decrease CREB protein expression, and CREB was only minorly reduced after DMGF treatment (Figure 8B). However, the results indicated that phosphorylation at Ser133 of CREB was suppressed by DMGF treatment (Figure 8B). These results revealed that DMGF could interfere with phosphorylation on the Ser133 of CREB which involves in the transcriptional activity. By this reaction, DMGF could decrease the activity of CREB to lower the expression of the genes that correspond to the formation of lamellipodia.

## DISCUSSION

DMGF is a bioactive biflavonoid from *Ouratea parviflora* [6] or *Taxus media* var. Hicksii [7]; however, little is known about its functions in tumor angiogenesis, metastasis and tumor growth except for its effect on inducing tumor death through apoptosis or autophagy in different cancer cells [7]. In this study, the DMGF was shown to dramatically suppress the spontaneous metastasis of B16F10 melanoma cells from subcutaneous sites to the spleen (Figure 4). Another two *in vivo* animal models further indicated that DMGF could suppress the processes of metastasis, including colonization from blood circulation (Figure 5) or invasion/intravasation into the lymph system (Figure 6). These results showed that the decrease in the metastasis of melanoma cells may result from low migratory ability (Figure 1B) because DMGF could reduce the formation of lamellipodia (Figure 7A) by interfering with F-actin polymerization by lowering the phosphorylation of CREB to suppress the expression of Cdc42/Rac pathway associated genes (Figures 7 and 8). This study is the first to show the medical potency of DMGF to suppress the metastasis of melanoma.

Cell migration is a key step for tumor metastasis. Some reports have demonstrated that certain flavonoids can suppress the migration of melanoma under different concentrations: quercetin at 60 μM (18 mg/ml) [16], (-)-epigallocatechin-3-gallate (EGCG) at 21.8 μM (10 mg/ml) [17], and genistein at 25 μM (6.8 mg/ml) [18] could cause similar effects on the inhibition of migration. Comparing the above information, only 1.8 μM (1 mg/ml) DMGF could have such the inhibitory effect on the migration of melanoma cells (Figure 1B). Thus, we thought that DMGF might have a higher potency than other flavonoids on the inhibition of migration.

Highly metastatic cancer cells frequently produce high levels of matrix metalloproteinases (MMPs) to degrade the extracellular matrix (ECM) surrounding them and to invade other tissues [19]. MMP-2 and MMP-9 are key enzymes for degrading type IV collagen, which is a major component of the basement membrane in ECM [14]. Figure 1C shows that DMGF has a stronger ability to suppress only MMP2 gene expression, but only a slight effect on MMP9 expression for B16F10 melanoma cells (Figure 1C). Flavonoids such as quercetin and EGCG have been shown to decrease the expression of both MMP2 and MMP9 in human melanoma cells [16] and breast cancer [20], respectively. These flavonoids inhibit the activities of both MMP2 and MMP9. However, 1 mg/ml DMGF only significantly suppressed the expression of MMP-2, but not MMP-9, at 12 h. Perhaps the dosages needed to inhibit the expression of MMP-2 or MMP-9 are different. The other possibility is that the mechanism by which DMGF suppresses MMP expression may be different from these flavonoids; it may suppress the signals needed by the MMP2 expression pathway.

The lamellipodium is a flat, sheet-like and F-actin-rich membrane protrusion, and it is the main organelle for cell locomotion. Tumor cells utilize this organelle to explore and move into the surrounding environment, then form invasive matrix-degrading structures [21]. In addition, the Rac1 or Cdc42 pathways can lead to the formation of membrane lamellipodia or filopodia, respectively, and have been shown to regulate a variety of biological functions, including changes in cell shape, adhesion, and motility, through the reorganization of the actin cytoskeleton [22, 23]. Recently, the literature demonstrated that some flavonoids could suppress such cell activity. For example, apigenin (50 μM) could influence F-actin cytoskeleton formation to suppress tumor cell motility [18]. Moreover, 30 μM luteolin inhibits the migration of human glioblastoma U-87 MG and T98G cells through the down-regulation of Cdc42 gene expression and ERK protein phosphorylation [24]. Moreover, 60 μM wogonin could suppress cell migration and actin remodeling of B16F10 cells by inhibiting the expression of Rac1 [25]. Here, the F-actin of the DMGF-treated B16F10 cells was remarkably disintegrated (Figure 7A), and the formation of lamellipodia was decreased to influence the cell's shape *in vitro* (Figure 7A). Sequentially, the expression of the relative genes in Cdc42/Rac1 pathways were examined, and the results showed that the expression of all relative genes was down-regulated by DMGF treatments (Figure 7B). Taken together, the decrease in the expressions of genes in Cdc42/Rac1 pathway may involve in the blockade of cell migration and invasion for DMGF's anti-metastatic activity as other flavonoids.

To identify which factors are involved in the DMGF-induced down-regulation of the genes, the transcriptional factor binding sites on the promoter sequences of these relative genes were assayed on GeneCards (http://www.genecards.org). Only one factor, CREB, was proposed to regulate the expression of all relative genes. Therefore, it was assumed that DMGF may reduce the phosphorylation or expression of CREB to down-regulate the expression levels of the genes related to the Cdc42/Rac1 pathway. Sequentially, our data fit with such suppositions. Figure 8A showed that DMGF treatments reduced the phosphorylation at Ser133 of CREB, but it did not influence the expression of CREB protein, which is in accord with the bioinformatics result which DMGF interacted with the amino acids near Ser133 of CREB (Figure 8B). These results indicated that the transcriptional activity of CREB would be decreased by DMGF. As previously reported, the metastatic phenotype of melanoma could benefit from the expression of CREB transcription factors [26]. Moreover, Xie et al. also demonstrated the decrease of CREB activity in metastatic melanoma cells by a dominant-negative form of CREB (KCREB), which led to a decrease in their tumorigenicity and metastatic potential in nude mice [27]. Moreover, a p21-activated kinase 4 (PAK4) inhibitor, PF-3758309, could suppress migration and invasion by reducing the phosphorylation of CREB and ERK1/2 [28]. Thus, DMGF down-regulates the phosphorylation of CREB to suppress the expression levels of these genes related to the Cdc42/Rac1 pathway, resulting in the inhibition of lamellipodia formation to attenuate metastasis.

In addition to lamellipodia formation, we proposed the other anti-metastatic mechanism for DMGF: the suppression of angiogenesis. Tumor angiogenesis could provide an escape route for those tumor cells that can loosen themselves from the primary tumor to new sites via new vessels [29]. Figure 3 shows *in vivo* DMGF treatment reduced the formation of new vessels and caused a decrease in the intensity of vessels in tumor areas. *In vitro*, DMGF could inhibit neovascularization by suppressing the motility of SVEC4-10 endothelial cells but not influencing the capillary-like tube formation in endothelial cells *in vitro* (Figure 2B and 2C). Therefore, we assume that DMGF could inhibit angiogenesis at early stages, and its anti-angiogenesis activity may also result from the blocking movement of the endothelial cells. Interestingly, the published literature also shows that two biflavonoids, amentoflavone (AF) and morelloflavone (MF), have anti-angiogenic activities. However, these reports only focus on the anti-angiogenic abilities of the two biflavonoids, but not their anti-metastatic effects. The anti-angiogenic activity of AF is due to its ability to bind VEGF-A and VEGF-B, preventing the interaction with VEGF receptors [5]; however, our data indicated that DMGF did not impact VEGF-induced capillary-like tube formation in SVEC4-10 endothelial cells (Figure 2B). Based on this different effect, we thought that DMGF may not bind to VEGF to prevent interactions with VEGF receptors for anti-angiogenesis. In addition, MF could inhibit the activation of Raf/MEK/ERK protein kinases and suppress the activation of AP-1 and p90RSK [11]. DMGF could suppress the phosphorylation of CREB, which is also an event downstream of ERK activation. Therefore, we assume that DMGF may inhibit both tumor angiogenesis by blocking the ERK/pCREB pathway in the same way as MF.

In the C57/BL6 mouse model, we found that DMGF at 10 mg/kg and 50 mg/kg significantly inhibited lung and lymph node metastasis, but did not affect the body weight of the mice (data not shown). DMGF inhibited the cell proliferation of SVEC4-10 as well as B16F10. SVEC4-10 is a highly proliferative endothelial cell line derived by SV40 transformation of endothelial cells. Therefore, SVEC4-10 is sensitive as B16F10 cells. However, in our previous results, DMGF had different antiproliferative effects between cancer cell lines and primary cells, including human PBMCs and mouse splenocytes [7]. These results showed that DMGF has selective cytotoxicity between normal and tumor cells. Thus, DMGF was assumed to be a novel chemoprevention drug with limited toxicity in normal tissues. Here, we first reported that DMGF has could suppress metastasis, including angiogenesis, due to its ability to suppress F-action polymerization by inhibiting the relative gene expression through the suppression CREB phosphorylation. Therefore, we propose that DMGF may be considered to be a potential chemoprevention drug for the inhibition of tumor metastasis.

## MATERIALS AND METHODS

### DMGF preparation

DMGF (98% purity by high performance liquid chromatography) isolated from the dried needles of *Taxus media* var. Hicksii was kindly provided by Yung-Shin Pharmaceutical Industry Co., Ltd. (Taichung, Taiwan) [7]. Briefly, the dried needles of *Taxus media* var. Hicksii (10 kg) were extracted with methanol (MeOH) at a preferred ratio of 1:1 (w/v) at room temperature to yield a methanol extract. The crude methanol extract was partitioned with water/ethyl acetate (EtOAc) (1:1), mixed with 350 g silical gel 60 N (Timely, Japan) for packing in an open column, and eluted with different ratios of hexane/EtOAc to afford 5 fractions (4:1, 3:1, 7:3, 13:7, 3:2, A–E). Fraction E was further purified by recrystallization from hexane/acetone (3:1) then re-chromatographed with the same elution condition to give 4.2 g (98.5 %) of DMGF sample.

### Cells and cell cultures

SVEC4-10 mouse vascular endothelial cells and B16F10 mouse melanoma cells were purchased from BCRC (Hsinchu, Taiwan, ROC) and maintained in Dulbecco's modified Eagle's medium (DMEM; Gibco/Invitrogen, Carlsbad, CA, USA) supplemented with heat-inactivated 10% fetal bovine serum (Gibco/Invitrogen, Carlsbad, CA, USA) and 1% penicillin/streptomycin/amphotericin in 5% CO_2_ at 37°C.

### Cell proliferation assay

The cells (1 × 10^4^ cells/well) were seeded overnight in a 96-well plate. After treatment with serial concentrations of DMGF in dimethyl sulfoxide (DMSO, final concentration of DMSO is 0.1%), the cells were incubated at 37°C for 48 h. Subsequently, the cell viability was measured by MTT assay. The cell viability ratio (%) was calculated using the following equation: % viability = absorbance of test sample/absorbance of control × 100%. The results were expressed in duplicate and for three independent experiments.

### Cell migration assay

For one set of experiments, SVEC4-10 cells were pre-cultured with 1 μg/ml DMGF for 6 h and then plated (3 × 10^4^ cells/well) in the upper well of a transwell plate (8 μm pore; Costar, Corning, NY, USA). In addition, B16F10 cells were pre-cultured with 1 μg/ml DMGF for 4 h (37°C, 5% CO_2_), then plated (3 × 10^4^ cells/well) in the upper well of a transwell plate. These cells were allowed to migrate toward the lower well with DMEM growth medium for 6 h (37°C, 5% CO_2_). Then, the cells on the top of the filter were removed with a cotton swab and the migrated cells on the underside were fixed with methanol, stained with 50 μg/ml propidium iodide, and counted with a fluorescence microscope.

### Tube formation assay

Fifty microliters of growth factor-reduced matrigel (BD Bioscience, San Jose, CA, USA) was added to each well of a 96-well plate and polymerized for 2 h at 37°C. SVEC 4–10 cells (3 × 10^4^) were treated with or without 1 μg/ml DMGF in growth medium containing 16 ng/ml of VEGF (Upstate Inc., Lake Placid, NY, USA). These cells were incubated for a further 4 h at 37°C and photographed under a microscope (100 × magnifications). The total number of network formations was counted.

### Real-time PCR

B16F10 cells (1 × 10^6^ cells/well) were treated with or without DMGF (1 μg/ml) for 12 or 24 h. Then, total B16F10 cellular RNA was extracted with TRIzol (Invitrogen Life Technologies, Carlsbad, CA, USA) and reverse-transcribed into cDNA using the SuperScript First-Strand Synthesis System (Invitrogen Life Technologies). The MMP2 and MMP9 mRNA levels were detected by real-time PCR (Applied Biosystems, Carlsbad, CA, USA). The primer pairs of MMP2, MMP9 and β-actin were as follows: 5′ CAC CTG GTT TCA CCC TTT CTG 3′ as the forward primer and 5′ AAC GAG CGA AGG GCA TAC AA 3′ as the reverse primer for MMP2*; 5′* GCT CAT GTA CCC GCT GTA TAG CT 3′ as the forward primer and *5′* CAG ATA CTG GAT GCC GTC TAT GTC 3′ as the reverse primer for MMP9. 5′ TTG CCG ACA GGA TGC CAG AA 3′ as the forward primer and 5′ GCC GACT CCA CAC GGA GTA CT 3′ as the reverse primer for mouse β-actin. The reaction mixture contained: 1 μl cDNA, 0.25 μl forward primer, 0.25 μl reverse primer, 11 μl DDW, and 12.5 μl 2X real Q PCR master mix (with 10 mM MgCl_2_, Green DNA dye). Real-time PCR consisted of a 95°C denaturation step for 10 min and followed by 40 cycles of 15 s at 95°C and 1 min at 60°C.

### Reverse transcription-polymerase chain reaction (RT-PCR)

Total cellular RNA was extracted with TriZol reagent (Life Technologies, Glasgow, UK) according to the manufacturer's instructions. Total RNA was reverse-transcribed using the Superscript™-III kit (Invitrogen, Carlsbad, CA). PCR analysis was performed on aliquots of the cDNA preparations to detect gene expression. The amplified products were visualized on 2% agarose gels. The PCR conditions were 4 min at 94°C followed by 30 cycles of 30 seconds at 95°C, 30 seconds at 55°C, and 1 min at 72°C. The following primers were used for the amplification reaction: 5′-GTG CCT GAG ATA ACT CAC CAC-3′ as the forward primer and 5′-TTC TGC ACT TAC ACA GAA AGG-3′ as the reverse primer for Cdc42; 5′-AAG ACA TCC AAC AGC CAG AA-3′ as the forward primer and 5′-TGT AGC CAC GTC CCG AGT-3′ as the reverse primer for PAK1; 5′-CCC TAT CCT ATC CGC AAA CA-3′ as the forward primer and 5′-CGC ACC TCA GGA TAC CAC TT-3′ as the reverse primer for Rac1; 5′-CCC CCA AAT GGT CCT AAT CT-3′ as the forward primer and 5′-ACA TGT CCA ATG TCT GGA A-3′ as the reverse primer for N-WASP1; 5′-GGG GCA TCA TCA AGA GCA-3′ as the forward primer and 5′-GAG GAC TAG GGT GGT TCAG-3′ as the reverse primer for LIMK; and 5′-CCA GCC GAG CCA CAT CGC TC-3′ as the forward primer and 5′-ATG AGC CCC AGC CTT CTC CAT-3′ as the reverse primer for GAPDH. The amplification was performed in 30 cycles of 50–60°C for 30 s, 72°C for 1 min, and 94°C for 30 s.

### Western blot

The cells were incubated for 12 h in the presence or absence of 2.5 μg/ml of DMGF. Then, the cells were collected and lysed in ice-cold RIPA lysis buffer. The protein concentration of the cell lysate was estimated with the Bradford protein assay using BSA as the standard. Total proteins (50−60 μg) were separated by SDS-PAGE using a 10% polyacrylamide gel and then transferred onto a nitrocellulose membrane. The membrane was blocked with 5% skim milk in phosphate buffered saline with Tween 20 (0.05% v/v Tween-20 in PBS, pH 7.2) for 1 h. The membranes were then incubated with anti-CREB antibody (1:1,000; GeneTex, Irvine, CA, USA) and anti-pCREB antibody (1:1000; Abcam, Cambridge, CA, USA) at 4°C overnight, followed by incubation with a horseradish peroxide-linked secondary antibody (1:10000; GeneTex, Irvine, CA, USA). The protein bands were visualized using the ChemiLucent ECL Detection System (Millipore, Billerica, MA, USA) and the Biospectrum AC Imaging System (UVP, Upland, CA, USA). The intensities of the chemiluminescence signal were quantified using the UVP VisionWorks LS Image Acquisition and Analysis Software (UVP, Upland, CA, USA).

### Confocal immunofluorescence

To observe the structures of lamellipodia, B16F10 cells plated on cover slides were incubated at 37°C overnight and then were treated by DMGF for 12 h. B16F10 cells were fixed with 4% formaldehyde for 10 min and were stained by phallotoxin (*Invitrogen*, Carlsbad, CA, *USA*) or ConA conjugated with Alexa Fluor 488 (*Invitrogen*, Carlsbad, CA, *USA*) for 60 min. The stained B16F10 cells were observed with 5 ml; of Hoest33342 mounting medium (Mounting Medium with Hoest33342, Abcam, Cambridge, CA, USA) by confocal microscopy.

### Anti-angiogenesis activity of DMGF *in vivo*

Female C57BL/6JNarl mice (8 weeks of age) were purchased from the National Laboratory Animal Center. All experiments with animals were performed in accordance with and approved by the Institutional Animal Care and Use Committee at National Chiao Tung University. Mice were inoculated subcutaneously with 1 × 10^6^ of B16F10 cells. Once a tumor mass of 30 mm^3^ was established, mice were intravenously injected with PBS, 10 mg/kg DMGF and 50 mg/kg DMGF once every three days. Mice were sacrificed on the final day of the experiments; tumors were removed and fixed by 4% paraformaldehyde, then examined by H&E staining to calculate the number of blood vessels. The vessel counts were counted with randomly ten fields (200× magnification) in each tumor tissue. The vessel numbers were considered as endothelial cells and countable microvessels morphology.

### Anti-lung metastatic activity of DMGF *in vivo*

The experimental metastasis model was established by the methods described previously [18]. B16F10 cells (1 × 10^6^ cells in 0.2 ml per mouse) were injected into C57BL/6JNarl mice via the lateral tail vein, and the mice were then randomly divided into different groups. Then, the mice were intravenously injected with DMGF at doses of 10 or 50 mg/kg, and the mice in the untreated group were injected with saline every 3 days. All of the mice were sacrificed on the 19^th^ day after tumor injection. Their lungs were then removed and fixed, the metastatic foci at the lung surfaces were photographed, and the number of tumor nodules on the lungs was calculated. All spleen tissues were collected and embedded in paraffin, and sections stained by H&E staining without melanin bleaching. The numbers of mtastatic melanoma cells were counted with randomly ten fields (400× magnification) in all spleen tissues. The percentages of melanoma cells were calculated as (melanin positive cells/total splenocytes and melanocytes)/ × 100% in each group.

### Anti-lymph nodes metastatic activity of DMGF *in vivo*

C57BL/6JNarl mice were inoculated *in situ* with 5 × 10^5^ B16F10-EGFP cells approximately 1 cm from the inguinal lymph node to directly establish experimental lymph nodes metastasis. Mice bearing metastatic cells were intravenously injected with saline, 10 mg/kg DMGF or 50 mg/kg DMGF once every three days. At day 9, the mice were sacrificed; lymph nodes were removed and embedded in O.C.T reagent. The frozen sections of lymph nodules were examined by fluorescence microscopy. Ten micrometer frozen sections of lymph nodes were stained with propidium iodide (PI) as a counter stain and analyzed with randomly ten fields (400× magnification) for each sample. The percentages of metastatic melanoma cells were calculated as [green fluorescence cells (GFP)/total red fluorescence cells (PI)] / × 100% in each group.

### CREB protein docking with DMGF

The docking of DMGF into CREB was explored using iGEMDOCK software [30–33], which have been shown to be powerful tools for molecular recognition and screening [34]. CREB1 (PDB code: 2LXT) was selected from Protein Data Bank (PDB) and DMGF was docked with it. The parameters of iGEMDOCK were setting such as population was set to 300, generation was set to 70 and solution was set to 100 [32].

### Statistical analysis

The results were analyzed using the SAS statistical software package (SAS Institute Inc., Cary, USA). The results were expressed as the means ± SD. Student's *t*-test was used when comparing two independent samples and ANOVA was used when comparing multiple samples. Differences with *p* < 0.05 were considered statistically significant.
